# Long-Term Efficacy of 5-ALA Photodynamic Therapy in Oral Lichen Planus Patients

**DOI:** 10.3390/ph18111676

**Published:** 2025-11-05

**Authors:** Magdalena Sulewska, Marta Wróblewska, Patryk Wiśniewski, Ewa Duraj, Jagoda Tomaszuk, Aleksandra Pietruska, Małgorzata Pietruska

**Affiliations:** 1 Department of Periodontal and Oral Mucosa Diseases, Medical University of Białystok, ul. Waszyngtona 13, 15-269 Białystok, Poland; magdalena.sulewska@umb.edu.pl (M.S.); patryk.wisniewski@umb.edu.pl (P.W.); ewa.duraj@umb.edu.pl (E.D.); jagoda.tomaszuk@umb.edu.pl (J.T.); 2 NZOZ“Trident” s. c., ul. Ks. Kard. Wyszyńskiego 16, 18-400 Łomża, Poland; darekimarta@gmail.com; 3 Student’s Research Group, Department of Periodontal and Oral Mucosa Diseases, Medical University of Białystok, ul. Waszyngtona 13, 15-269 Białystok, Poland; perio@umb.edu.pl

**Keywords:** oral lichen planus, 5-aminolevulinic acid, ALA-PDT, photodynamic therapy, long-term outcomes, VAS, REU

## Abstract

**Background:** Oral lichen planus (OLP) is a chronic mucosal disease associated with a risk of malignant transformation. Although topical corticosteroids are the standard therapy, prolonged administration may result in local and systemic complications. Photodynamic therapy (PDT) with 5-aminolevulinic acid (ALA) has been proposed as a less invasive and safer alternative. **Methods:** In this prospective study, 44 patients with histologically verified OLP underwent a protocol consisting of ten consecutive weekly PDT sessions, each comprising a single irradiation. A 5% ALA formulation was topically applied, followed by illumination with a 630 nm red light device. Clinical outcomes were evaluated at baseline, immediately after therapy, and at 12- and 48-month follow-ups. Changes in lesion surface, REU index, and pain intensity on a visual analog scale (VAS) were analyzed. **Results:** Significant improvements were noted, with progressive and sustained decreases in lesion extent, REU scores, and VAS values throughout the 4-year observation period. The therapeutic response was consistent across different mucosal sites (keratinized and non-keratinized). No treatment-related adverse reactions were recorded. **Conclusions:** Long-term follow-up indicates that ALA-mediated PDT is a safe and effective management option for both reticular and erosive variants of OLP. Its durable clinical benefits and favorable safety profile support its role as an alternative to corticosteroid therapy.

## 1. Introduction

Oral lichen planus (OLP) is a chronic immune-mediated inflammatory disease, classified among oral potentially malignant disorders (OPMDs), with an estimated malignant transformation risk of 1.1–2.28%, lowest in the reticular form [1]. It presents in various clinical forms and is often accompanied by burning sensations, pain, and discomfort, which considerably impair patients’ quality of life [2,3].

The pathogenesis of OLP involves a complex interplay of immune dysregulation, genetic predisposition, infectious agents, and environmental or iatrogenic factors [4,5,6,7,8,9,10,11]. Among these, cytotoxic T-cell activity and Th1/Th17-driven inflammation play a central role in epithelial damage [4,5].

Topical glucocorticosteroids (GCSs) remain the gold standard in OLP management, but their long-term use is limited by adverse effects such as mucosal atrophy, fungal superinfections, and treatment resistance [10,11,12,13].

Photodynamic therapy (PDT) has emerged as an alternative, non-invasive treatment. It combines a photosensitizer, light of an appropriate wavelength, and oxygen, generating reactive oxygen species that selectively destroy diseased cells while sparing healthy tissues [14,15,16,17,18,19]. In addition to direct cytotoxicity, PDT may also impair local vasculature and stimulate immune responses [20,21]. It has been successfully used for precancerous lesions, skin cancers, and chronic oral mucosal conditions, including OLP [22,23].

Importantly, PDT has also been applied in OLP with epithelial dysplasia. Shang et al. (2020) reported successful management of OLP with moderate-to-severe dysplasia, while Jerjes et al. (2011) and Binnal et al. (2022) confirmed its effectiveness in oral epithelial dysplasia and other OPMDs, supporting its role in the management of lesions with malignant potential [24,25,26].

Among available photosensitizers, 5-aminolevulinic acid (5-ALA) is one of the most widely used. Following topical application, it is metabolized to protoporphyrin IX, which accumulates in abnormal cells and can be activated by red light, leading to selective cytotoxicity [27,28,29,30]. However, its relatively low lipophilicity and rapid metabolism may reduce efficacy, while the penetration depth of red light restricts application to superficial lesions [31,32].

Although several studies have shown promising outcomes with PDT in OLP, evidence on long-term effectiveness remains limited. Therefore, the aim of the present study was to evaluate the clinical effectiveness of photodynamic therapy using a proprietary 5% 5-ALA formulation in the treatment of OLP during a four-year follow-up.

## 2. Results

A total of 44 patients diagnosed with oral lichen planus (OLP) were included in the study. The majority were women (77.3%), and multifocal lesions were observed in 88.6% of the cases. The reticular form was more prevalent (84.09%) than the erosive form (15.91%). In total, 110 lesions were analyzed: most were located on non-keratinized oral mucosa (71.82%), and 88.18% were reticular in form (Table 1).

### 2.1. Lesion Size

A statistically significant reduction in lesion dimensions was observed in the total group (*p* < 0.0001). The mean lesion area decreased from 4.05 ± 3.86 mm^2^ at baseline (T0) to 0.67 ± 1.43 mm^2^ at T3. A similar trend was noted in both clinical forms. In the reticular form (*n* = 94), the lesion area decreased from 4.47 ± 3.99 mm^2^ at T0 to 0.77 ± 1.53 mm^2^ at T3 (*p* < 0.0001). In the erosive form (*n* = 16), a substantial reduction from 1.60 ± 1.51 mm^2^ at T0 to 0.10 ± 0.21 mm^2^ at T3 was also statistically significant (*p* < 0.0001) (Table 2).

### 2.2. Subjective Symptoms—VAS Score

Analysis of the Visual Analog Scale (VAS) showed a statistically significant reduction in subjective symptoms (*p* < 0.0001). In the total group, the median score decreased from 4.00 (IQR: 2.00–6.00) at T0 to 1.00 (IQR: 0.00–2.00) at T3. Similar results were recorded in patients with the reticular form (*n* = 37), where the VAS score dropped from 4.00 to 1.00 (*p* < 0.0001). In the erosive form group (*n* = 7), the median value decreased from 4.00 to 2.00 (*p* = 0.0216) (Table 3).

### 2.3. Clinical Index—REU Score

The REU index also showed a significant decline during follow-up. In the total group (*n* = 44), the score decreased from 4.86 ± 3.46 at T0 to 1.68 ± 1.83 at T3 (*p* < 0.0001), indicating notable clinical improvement (Table 4).

### 2.4. Healing Rates

Analysis of healing distribution based on lesion location and clinical form revealed that lesions on non-keratinized mucosa showed a slightly lower healing rate (58.23%) compared to keratinized mucosa (61.29%). In the reticular form group, 57.97% of lesions on non-keratinized mucosa and 60.00% on keratinized mucosa were healed. Among erosive lesions, 60.00% of non-keratinized and 66.67% of keratinized lesions showed complete healing. No correlation was found between lesion healing and their localization on the oral mucosa (keratinized vs. non-keratinized) (*p* > 0.05) (Table 5).

## 3. Discussion

Oral lichen planus (OLP) poses a significant therapeutic challenge due to its chronic nature, tendency to recur, and considerable subjective symptoms reported by patients [1,2,3]. Despite the availability of numerous proposed treatment modalities, a universally effective and standardized therapeutic approach remains lacking. Conventional treatment, primarily based on the topical use of corticosteroids, has proven effective in alleviating symptoms, but carries the risk of adverse effects, including mucosal atrophy, fungal infections, and taste disturbances [10,12]. As a result, photodynamic therapy (PDT) has been attracting growing attention, with both our findings and numerous literature reports supporting its efficacy and safety. The main clinical indications for PDT include lesions resistant to conventional corticosteroid therapy as well as cases in which the use of steroids is contraindicated due to systemic conditions or patient-specific risk factors [33].

In our study, the PDT protocol based on 5-aminolevulinic acid (ALA) and red light (630 nm) resulted in a significant reduction in lesion area, particularly in the reticular form. Improvement was observed both immediately after treatment and at 12- and 48-month follow-ups, confirming the long-term therapeutic effect. Similar results were reported by Kvaal et al. (2013), who used a single session of PDT with methyl aminolevulinate (MAL) and noted clinical improvement after 6 months, with sustained effects after 4 years [34].

Maloth et al. (2016) compared PDT and corticosteroid therapy in OLP patients, demonstrating significant lesion size reduction in both groups, with greater efficacy observed in the PDT group (reduction from 2.22 cm^2^ to 1.41 cm^2^ vs. 2.27 cm^2^ to 1.74 cm^2^) [35]. Recent randomized clinical trials have confirmed this trend: Sulewska et al. (2025) reported a greater decrease in lesion size after PDT compared with corticosteroids, while Zborowski et al. (2025) demonstrated that MB-PDT produced lesion shrinkage superior to clobetasol [36,37]. Our findings confirm this trend, with even more pronounced reduction (from 4.05 cm^2^ before treatment to 0.67 cm^2^ at 48 months).

The literature also includes comparisons of various PDT protocols. Bakhtiari et al. (2017) employed methylene blue and LED light, finding that PDT was comparable in efficacy to dexamethasone [38]. Mostafa et al. (2017) reported even better outcomes, with PDT providing greater clinical improvement than conventional treatment [39]. The authors emphasized the importance of physiological factors such as tissue oxygen availability and vascular response, which may influence therapeutic outcomes [39].

Mirza et al. (2018), in their comparison of PDT, low-level laser therapy (LLLT), and corticosteroids, also demonstrated superior efficacy of PDT in reducing OLP lesions [40]. More recently, Salinas-Gilabert et al. (2023) compared PDT, photobiomodulation, and topical corticosteroids, showing significant clinical improvement and VAS reduction in the PDT group, further confirming the versatility of this method [41]. Our findings support this, confirming the effectiveness of PDT regardless of the protocol used.

An interesting aspect of therapy effectiveness is the role of lesion location. Sobaniec et al. (2013) observed significant reduction in lesions on non-keratinized mucosa [42]. Similar findings were reported in a meta-analysis by He et al. (2020), although the differences did not reach statistical significance [33]. In our study, despite initially larger lesions on non-keratinized mucosa, a significant reduction was observed regardless of location.

The REU index, used to assess clinical severity of OLP, significantly decreased in our study (from 4.86 to 1.68). Comparable results were reported by Rakesh et al. (2018), who used ALA-based PDT [43]. These results are in line with Sulewska et al. (2025), who demonstrated a marked reduction in both REU and VAS after PDT, and with the systematic reviews by Gulzar et al. (2023) and Nagi et al. (2023), which highlighted consistent improvements in REU, Thongprasom score, and pain intensity across multiple RCTs [36,44,45].

In terms of pain reduction measured by the Visual Analog Scale (VAS), our PDT protocol showed high efficacy. These results are consistent with findings by Lavaee et al. (2019) and the meta-analysis by He et al. (2020), which reported a mean VAS reduction of 3.82 points [33,46]. Furthermore, Mostafa et al. (2017) confirmed the superiority of PDT over corticosteroids in reducing pain in erosive OLP [39]. Similarly, Zborowski et al. (2025) and Salinas-Gilabert et al. (2023) observed significant reductions in VAS after PDT, supporting its role as a pain-relieving therapy [37,41].

Romano et al. (2021) and Sulewska et al. (2019) both confirmed the effectiveness of PDT in treating both reticular and erosive forms of OLP [47,48]. In our study, erosive lesions also showed reduction over time, particularly on keratinized mucosa.

Taken together, our findings are consistent with most literature reports. PDT based on ALA and 630 nm light was shown to be effective in improving both subjective symptoms and objective clinical signs of OLP [49,50]. The absence of adverse effects, good tolerability, and long-lasting results support the use of this method as a valuable alternative to conventional corticosteroid therapy.

In the management of OLP, the exact mechanisms underlying the effects of PDT are not yet fully elucidated. It is believed that, apart from the aforementioned pathways, PDT may also modulate immune signaling by stimulating the expression of pro-inflammatory cytokines, such as IL-1 and IL-6 [51]. Light activation induces a transient increase in these mediators, leading to a short-term amplification of the inflammatory response, enhanced generation of reactive oxygen species (ROS), and apoptosis of abnormal cells. Moreover, Cosgarea et al. (2020) demonstrated that PDT decreases the proportion of CD137+ activated T lymphocytes, thereby reducing chronic inflammation [52]. PDT has also been reported to reduce oxidative stress, a factor regarded as crucial in the pathogenesis and chronic course of OLP. Wiśniewski et al. (2025) confirmed this effect, demonstrating a significant decrease in salivary oxidative stress markers (TOS, TAC, OSI) after PDT, thus providing biochemical evidence of its antioxidative and immunomodulatory action [53].

In addition to the biological mechanisms underlying the therapeutic effect of PDT, formulation-related aspects should also be considered. Another factor that may influence the therapeutic efficacy of oral formulations used in OLP is the role of excipients. Chiriac et al. (2016) demonstrated that excipients significantly affect the physical characteristics and pharmaceutical performance of oral lyophilisates containing a pregabalin–acetaminophen combination, particularly in terms of disintegration time and drug release profile [54]. These findings highlight that, beyond the active compound itself, formulation components can modulate the overall effectiveness of oral dosage forms [54].

The major limitation of this study is the absence of a control group. This decision was primarily dictated by ethical considerations, as all enrolled patients were symptomatic and leaving them untreated or assigning them to a placebo arm was not justifiable. Moreover, the study was designed as a prospective clinical investigation rather than a randomized controlled trial (RCT). Therefore, direct comparison with other therapeutic modalities was beyond its scope and would have required a different design, with additional methodological and regulatory demands as well as considerably higher costs. Other limitations include the relatively small sample size, which, despite the long follow-up period, may restrict the generalizability of the findings. In addition, the complexity of the protocol, involving ten weekly PDT sessions, may pose challenges for routine clinical implementation. To address these issues, efforts have been directed toward developing a novel 5-ALA-based emulgel with improved mucoadhesive properties (patent application P.443813, PL), the evaluation results of which will soon be published by our group. Nevertheless, recent randomized studies [36,37] have begun to address the question of comparability, while systematic reviews [44,45] underline the need for larger multicenter RCTs using standardized indices such as REU and VAS.

Despite the promising outcomes, further studies are necessary to optimize PDT protocols and standardize clinical evaluation methods. Future indications for PDT may include patients with contraindications to glucocorticosteroids, lesions unresponsive to conventional steroid therapy, and multifocal OLP, where simultaneous application of the photosensitizer and sequential irradiation of multiple sites can be effectively performed.

## 4. Materials and Methods

The investigation was designed as a prospective controlled clinical study. A total of 44 patients aged 30–88 years (mean: 58.5), including 34 women and 10 men, were enrolled in the study. All participants reported to the Department of Periodontal and Oral Mucosa Diseases at the Medical University of Białystok for treatment of oral lesions between April 2017 and August 2019. The study was approved by the Bioethics Committee of the Medical University of Białystok (approval no. R-I-002/102/2017). The trial was conducted in accordance with the principles of the Declaration of Helsinki, and the reporting followed the TREND (Transparent Reporting of Evaluations with Nonrandomized Designs) statement guidelines. This study was retrospectively registered in DRKS (Registry name: DRKS; Registration numberDRKS000038334; Date of registration:3 November 2025). Registration was performed retrospectively, as the trial was initiated in 2017 before clinical trial registration was mandated by local regulations.

Inclusion criteria comprised histopathologically confirmed oral lichen planus and age over 18 years. The diagnostic framework for oral lichen planus (OLP) remains somewhat variable, as the criteria have been refined and modified in recent years. Current expert consensus recommends that the diagnosis should combine both clinical presentation and histopathological evidence. From a clinical perspective, characteristic features include: (1) bilateral, symmetrical white lesions most commonly affecting the buccal mucosa, but also the tongue, lips, or gingiva; (2) the presence of whitish papules or slightly elevated striae on the mucosa, which may coexist with erosions; and (3) in some cases, desquamative gingivitis as an accompanying manifestation. Histopathological hallmarks are: (1) a dense, band-like lymphocytic infiltrate localized in the superficial lamina propria; (2) vacuolar degeneration within the basal cell layer and/or suprabasal region, accompanied by keratinocyte apoptosis; and (3) epithelial atrophy or ulceration associated with a mixed inflammatory infiltrate, particularly in the atrophic/erosive variant [55]. For the purposes of this study, only patients with the reticular or erosive clinical subtypes were included, and none of the examined specimens revealed epithelial dysplasia. Exclusion criteria included systemic diseases that may present with oral lesions, smoking, pregnancy or lactation, and treatment of oral lesions within the previous six months.

### 4.1. Therapeutic Procedure

Treatment was based on a proprietary PDT protocol using 5% 5-aminolevulinic acid (ALA) (Ala-Plus, Farmapol, Poznań, Poland) as a photosensitizer. The gel was applied to the saliva-dried lesion and surrounding oral mucosa (approx. 2 mm layer) two hours before irradiation. It was protected with an occlusive dressing made of nonwoven fabric that extended beyond the lesion margins and was secured with multiple layers of sterile gauze. The application was repeated four times at 30 min intervals. In cases of multifocal OLP, the photosensitizer was applied simultaneously to all lesions, and after the incubation period each site was sequentially irradiated for the appropriate time. Two hours after the first application, the OLP lesion was irradiated with a custom-designed LED lamp delivering light at a wavelength of 630 nm and a power output of 300 mW from the fiber-optic tip. The irradiation was performed in a non-contact, continuous-wave mode, with a surface energy density of 120 J/cm^2^. PDT sessions were repeated ten times at weekly intervals.

### 4.2. Clinical Examination

**Figure 1:**
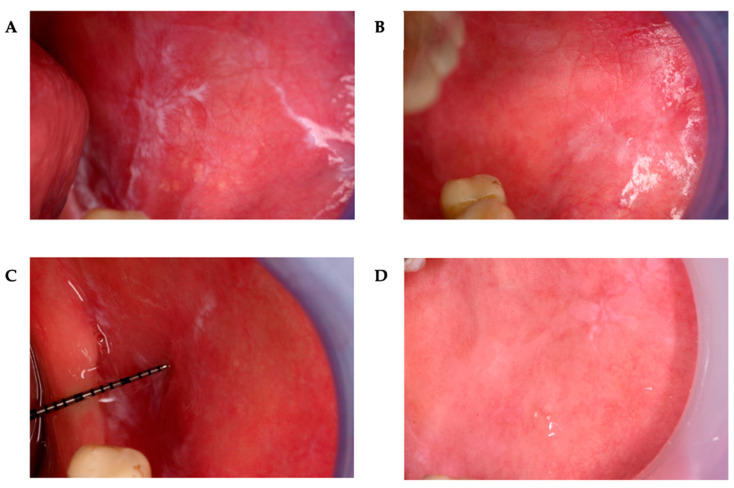
Reticular form of OLP lesion on the buccal mucosa. (**A**) before treatment (T0), (**B**) immediately after treatment (T1), (**C**) one year after treatment (T3), and (**D**) four years after treatment (T4).

**Figure 2:**
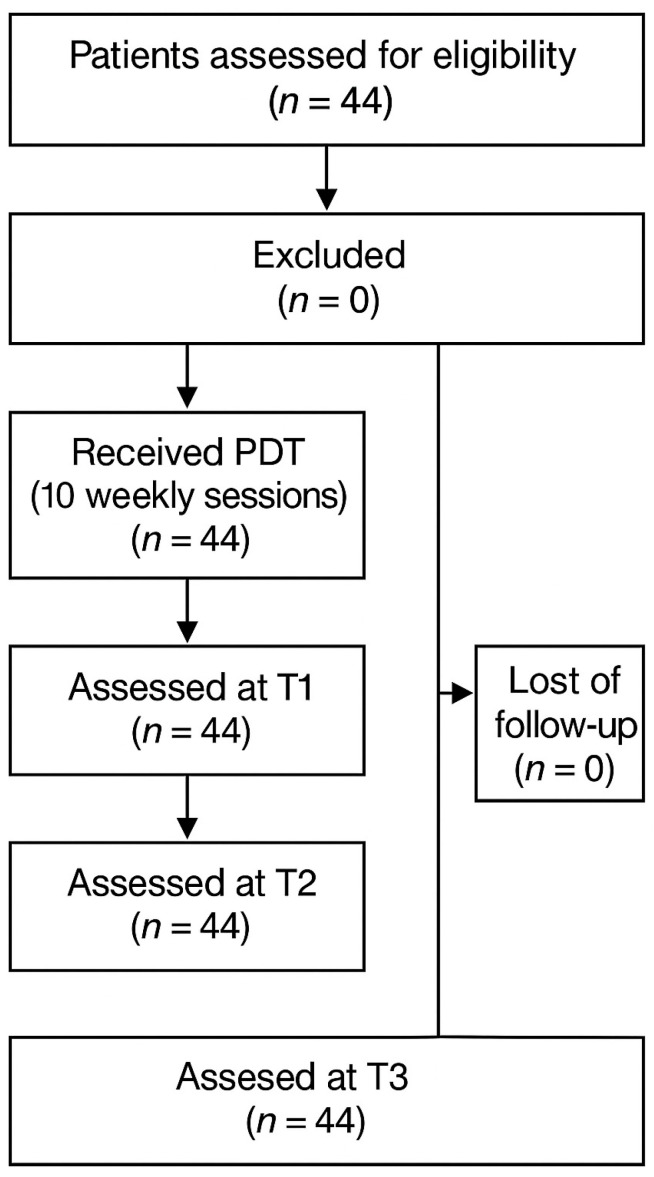
Patient’s flow chart.

All clinical measurements were performed by the same examiner to ensure consistency of assessment. The clinical assessment included evaluation of the location and surface area of OLP lesions using a PCPUNC15 periodontal probe (Hu-Friedy, Chicago, IL, USA). The examination was performed before treatment (T0), immediately after treatment (T1), 12 months post-treatment (T2), and 48 months post-treatment (T3). Lesions were categorized by size as follows: no visible lesions, ≤1 cm^2^, >1 cm^2^ and ≤3 cm^2^, and >3 cm^2^ (Figure 1 and Figure 2).

To assess lesion severity, the REU scoring system (Reticulation, Erythema, Ulceration) was used. Lesions were scored as follows: reticular lesions—R (0 = absent, 1 = present); erythematous/erosive lesions—E, and ulcerative lesions—U (0 = absent, 1 = <1 cm^2^, 2 = 1–3 cm^2^, 3 = >3 cm^2^). The total REU score was the sum of the points for the presence of reticular, erythematous, and ulcerative changes [56].

Additionally, patients completed a questionnaire regarding subjective symptoms in the oral cavity. The intensity of pain, burning, and itching was assessed using the Visual Analog Scale (VAS), where 0 = no symptoms, 1–3 = mild, 4–6 = moderate/severe, 7–9 = very severe, and 10 = worst imaginable pain [57].

### 4.3. Statistical Analysis

Sample size estimation was performed using the G*Power 3.1 software, assuming a statistical power of 80% and a significance level of 0.05. The analysis indicated that the minimum required sample size was 28 participants for both the repeated-measures ANOVA and the Friedman test. The characteristics of the lesion size groups were presented as counts and percentages. Quantitative variables were described using means with 95% confidence intervals, standard deviations, medians, lower and upper quartiles, and minimum and maximum values. Normality of data distribution was verified with the Shapiro–Wilk test. In case of significant deviation from normal distribution, the Friedman test and Dunn’s multiple comparison test with Bonferroni correction or the Mann–Whitney U test were used, depending on the number of compared groups. If data followed a normal distribution and the assumption of sphericity was violated, ANOVA and Fisher’s LSD post hoc test were applied. Nominal-scale variables were analyzed using the chi-square test or Fisher’s exact test. The significance level was set at α = 0.05. All analyses were performed using Statistica software (version 13) and PQStat software (version 1.6.6).

## 5. Conclusions

OLP is a chronic inflammatory condition classified as an OPMD, which necessitates therapeutic strategies that are both effective and safe. The findings of this study confirm that PDT with 5-ALA may serve as a promising minimally invasive approach for managing both reticular and erosive forms of OLP. The absence of adverse effects, good tolerability, sustained clinical improvement—including lesion size reduction, pain relief, and REU score normalization—and enhanced quality of life support the potential of PDT in the long-term management of OPMDs. These results represent an important step toward the development of non-invasive therapeutic standards for precancerous oral conditions. Further studies on larger cohorts are warranted to validate and standardize PDT protocols.

## Figures and Tables

**Table 1:** Demographic and clinical characteristics of the study group, including the number and type of lesions, lesion localization, and lesion morphology.

**Variable**	**Number of patients (*n*)**	**Percentage (%)**
Sex		
Women	34	77.3%
Men	10	22.7%
Number of lesion sites		
Single-site lesions	5	11.4%
Multifocal lesions	39	88.6%
Clinical form of lesions		
Erosive form	7	15.91%
Reticular form	37	84.09%
**Variable**	**Number of lesions (*n*)**	**Percentage (%)**
Lesion localization		
Keratinized oral mucosa	31	28.18%
Non-keratinized oral mucosa	79	71.82%
Clinical form of lesions		
Erosive form	16	11.82%
Reticular form	94	88.18%

**Table 2:** Changes in lesion area (cm^2^) over time in the total study group and in reticular and erosive subtypes of OLP. Results are presented as mean ± standard deviation (SD), 95% confidence intervals (CI), and range (Min–Max). Statistical significance was assessed using Friedman test.

Group	Time Point	Number of Lesions	Mean ± SD	95% CI	Min–Max	Friedman Test (*p*)
Total group	T0	110	4.05 ± 3.86	3.32–4.78	0.09–18.00	<0.0001
	T1		1.51 ± 1.98	1.13–1.88	0.00–9.24	
	T2		0.69 ± 1.39	0.42–0.95	0.00–9.36	
	T3		0.67 ± 1.43	0.40–0.94	0.00–8.20	
Reticular	T0	94	4.47 ± 3.99	3.66–5.29	0.24–18.00	<0.0001
	T1		1.49 ± 1.99	1.08–1.90	0.00–9.24	
	T2		0.78 ± 1.47	0.47–1.08	0.00–9.36	
	T3		0.77 ± 1.53	0.45–1.08	0.00–8.20	
Erosive	T0	16	1.60 ± 1.51	0.79–2.41	0.09–5.00	<0.0001
	T1		1.62 ± 1.98	0.57–2.68	0.00–5.67	
	T2		0.18 ± 0.53	−0.09–0.46	0.00–2.10	
	T3		0.10 ± 0.21	−0.01–0.21	0.00–0.81	

**Table 3:** Descriptive statistics of lesion count across time points in the total study group and in reticular and erosive forms of OLP. Values include median, minimum, maximum, lower and upper quartiles. Statistical significance was determined using the Friedman test.

Group	Time Point	*n*	Median	Min	Max	Lower Quartile	Upper Quartile	Friedman Test (*p*)
Total group	T0	44	4.00	0.00	9.00	2.00	6.00	<0.0001
	T1		2.00	0.00	8.00	1.00	4.00	
	T2		1.00	0.00	6.00	0.00	3.00	
	T3		1.00	0.00	6.00	0.00	2.00	
Reticular form	T0	37	4.00	0.00	9.00	1.00	6.00	<0.0001
	T1		2.00	0.00	8.00	1.00	4.00	
	T2		1.00	0.00	6.00	0.00	3.00	
	T3		1.00	0.00	6.00	0.00	2.00	
Erosive form	T0	7	4.00	2.00	7.00	4.00	6.00	0.0216
	T1		3.00	0.00	5.00	2.00	4.00	
	T2		2.00	0.00	5.00	0.00	3.00	
	T3		2.00	1.00	4.00	1.00	4.00	

**Table 4:** Changes in REU index scores across follow-up time points (T0–T3) in the total study group. Values are presented as mean ± standard deviation (SD), 95% confidence intervals (CI), and range (Min–Max). Statistical significance assessed using the Friedman test.

Time Point	*n*	Mean	SD	95% CI (Lower)	95% CI (Upper)	Min	Max	Friedman Test (*p*)
T0	44	4.86	3.46	3.81	5.91	1.00	19.00	<0.0001
T1		3.72	2.89	2.84	4.60	0.00	11.00	
T2		2.93	2.67	2.11	3.74	0.00	9.50	
T3		1.68	1.83	1.12	2.24	0.00	8.00	

**Table 5:** Relationship between lesion localization (keratinized vs. non-keratinized oral mucosa) and healing status at T3 in the total group and subgroups (reticular and erosive forms). Results of the Chi-square test are provided for each comparison.

Group	Healed/Not Healed	Non-Keratinized Mucosa *n* (%)	Keratinized Mucosa *n* (%)	Chi-Square Test *p*
Total	Healed	46 (58.23%)	19 (61.29%)	0.7688
	Not healed	33 (41.77%)	12 (38.71%)	
Reticular form	Healed	40 (57.97%)	15 (60.00%)	0.86
	Not healed	29 (42.03%)	10 (40.00%)	
Erosive form	Healed	6 (60.00%)	4 (66.67%)	1.00
	Not healed	4 (40.00%)	2 (33.33%)	

## Data Availability

The original contributions presented in this study are included in the article. Further inquiries can be directed to the corresponding author.
